# Initial Experience of Non-Atriotomy Surgical Ablation During Coronary Artery Bypass Grafting With Preexisting Atrial Fibrillation: A Multicenter Study

**DOI:** 10.1016/j.atssr.2025.10.010

**Published:** 2025-11-13

**Authors:** Armin Kiankhooy, Gregory Rushing, Marc Pelletier, J. Hunter Mehaffey, Manuel Gomez-Tschrnko, Marc Gerdisch, Andrew Barksdale, Manesh Parikshak, Joshua Chung, Yingyot Arora, Gianluca Torregrossa, Massimo Baudo, Isaac George, Fady Soliman

**Affiliations:** 1 Department of Cardiac Surgery, Smidt Heart Institute, Cedars-Sinai Medical Center, Los Angeles, California; 2 Department of Cardiac Surgery, The Heart Surgery Center of University Hospitals Harrington Heart & Vascular Institute, Cleveland, Ohio; 3 Department of Cardiac Surgery, Yale School of Medicine, New Haven, Connecticut; 4 Department of Cardiovascular and Thoracic Surgery, West Virginia University, Morgantown, West Virginia; 5 Department of Cardiothoracic Surgery, Franciscan Health, Indianapolis, Indiana; 6 Department of Cardiothoracic Surgery, Loma Linda University, Loma Linda, California; 7 Department of Cardiac Surgery, Lankenau Heart Institute, Mainline Health, Wynnewood, Pennsylvania; 8 Department of Surgery, NewYork-Presbyterian/Columbia University Medical Center, New York, New York

## Abstract

**Background:**

Guideline concordance with surgical ablation in patients undergoing isolated coronary artery bypass grafting (CABG) is poor. Additional atriotomy and safety concerns are barriers to ablation. We sought to evaluate the initial safety and associated rhythm outcomes of a novel non-atriotomy surgical ablation (NASA) left atrial box lesion in patients undergoing isolated CABG.

**Methods:**

A multicenter retrospective review included all concomitant NASA in patients with preexisting atrial fibrillation undergoing isolated CABG. The primary outcome was NASA-related intraoperative complications. Associated rhythm outcomes were per the Heart Rhythm Society definition of freedom from atrial fibrillation, atrial flutter, and atrial tachycardia (FFAF) <30 seconds at 12 months off class I or III antiarrhythmic medications by >24-hour continuous monitoring. Data are reported as median (interquartile range).

**Results:**

Ninety-seven patients were analyzed. The median age was 73 (68-76) years; CHA_2_DS_2_-Vasc score, 4.0 (3-5); left ventricular ejection fraction, 55% (45%-60%); and left atrial diameter, 4.0 (3.6-4.7) cm. Most patients (86% [83/97]) had paroxysmal atrial fibrillation. The primary safety outcome of intraoperative complications attributed to NASA was not observed (0% [0/97]). Thirty-five patients (35/97 [36%]) had at least 12 months (median, 12 months; interquartile range, 12-24 months) of continuous ambulatory monitoring, and FFAF was 94% (33/35); 91% (29/32) showed FFAF off antiarrhythmic medications.

**Conclusions:**

In patients with mostly paroxysmal atrial fibrillation undergoing isolated CABG surgery, concomitant NASA was safe and associated with favorable restoration of normal sinus rhythm at 1 year.

**In Short**

▪ In a multicenter real-world analysis, a non-atriotomy surgical ablation (NASA) with an all-encompassing bipolar radiofrequency clamp is safe.
▪ NASA of the left atrium in patients with paroxysmal atrial fibrillation undergoing isolated coronary artery bypass grafting was associated with freedom from atrial fibrillation off antiarrhythmic drugs with at least 12-month follow-up.

Concomitant surgical ablation at the time of coronary artery bypass grafting (CABG) has been supported with a class I recommendation since the initial Society of Thoracic Surgeons (STS) 2017 Clinical Practice Guidelines for the Surgical Treatment of Atrial Fibrillation^1^ and again in the 2023^2^ update. Despite these recommendations, guideline concordance continues to be poor, with a 31% surgical ablation rate in patients undergoing isolated CABG in 2022.^2^ Barriers to ablation at CABG may include patient safety concerns and perceived technical complexity of performing surgical ablation by atriotomy. Recent STS-linked Medicare analysis has revealed that most (80%) cardiac surgical patients present with paroxysmal atrial fibrillation (PAF) and only 20% present with more advanced non-PAF (persistent and long-standing persistent).^3^ Patients with PAF are hypothesized to have a trigger-based mechanism of atrial fibrillation that predominantly originates in the pulmonary veins^4^ and left atrial posterior wall.^5^ Therefore, surgical ablation targeting only these triggers may provide an effective solution for most CABG patients with atrial fibrillation and possibly improve guideline concordance. In August 2021, a novel bipolar radiofrequency clamp was introduced that allows a non-atriotomy surgical ablation (NASA) of bilateral pulmonary veins and the left atrial posterior wall or left atrial “box” lesion.^6^ A paucity of evidence currently exists evaluating the safety and efficacy of this approach.^7^ We sought to evaluate the safety and efficacy of a NASA approach in patients with preexisting atrial fibrillation undergoing isolated CABG.

## Patients and Methods

### Study Design and Population

We performed a retrospective review of all consecutive patients undergoing isolated CABG surgery treated from July 1, 2023, until December 30, 2024. There were 153 patients identified with preexisting atrial fibrillation undergoing first-time isolated CABG who underwent surgical ablation with the novel bipolar radiofrequency clamp; 22 patients underwent NASA and additional linear ablations by atriotomy, so these cases were excluded, leaving a new total of 131 patients. Also excluded were 34 patients who had <2 months (“blanking period”) of clinical rhythm follow-up, leaving a new total of 97 patients for initial analysis. Further analysis was carried out on 43 patients who had continuous ambulatory monitoring (CAM) and 35 patients who had CAM for at least 12 months.

### Rhythm Monitoring

Patients were monitored for post-NASA freedom from atrial fibrillation, atrial flutter, and atrial tachycardia (FFAF) by standard electrocardiography or CAM. When CAM was available, the Heart Rhythm Society^8^ definition of <30 seconds of atrial fibrillation, atrial flutter, or atrial tachycardia was analyzed and determined to define rhythm success. At last follow-up, there were 54 patients (54/97 [55.7%]) who had electrocardiography only, whereas 43 (43/97 [44.3%]) had CAM rhythm assessment. Standard clinical outcomes data were retrieved by review of the center-specific electronic medical records and STS collected data.

**Figure 1:**
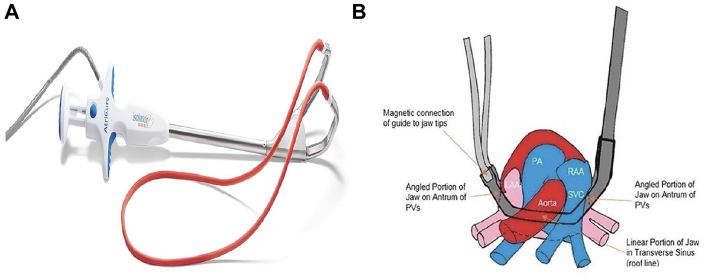
(A) The EnCompass device (AtriCure, Inc) is a nonirrigated dual-electrode bipolar radiofrequency clamp. The magnetized red rubber guide system is used to assist in the placement of the clamp. (B) The EnCompass clamp creates a box lesion that isolates the entire left atrial posterior wall in a single application. (LAA, left atrial appendage; PA, pulmonary artery; PVs, pulmonary veins; RAA, right atrial appendage; SVC, superior vena cava.) Reprinted from^6^ with permission.

NASA was performed with the Isolator Synergy EnCompass device (AtriCure, Inc), a 510(k)-approved dual-electrode bipolar radiofrequency nonirrigated clamp with a 105-mm electrode length for cardiac tissue ablation (Figure 1). The clamp is positioned around the posterior left atrium through the oblique and transverse cardiac sinuses with a magnet-tipped red rubber catheter loop. Ablation protocol included a minimum of 3 pairs of transmural ablations as depicted on the visual monitor of the radiofrequency generator device. All patients had left atrial appendage occlusion.

### Statistics

Statistical analysis was performed with Prism 9.2.0 (GraphPad Software), and figures were created with Microsoft Excel 16.77.1. Variables are reported as number (percentage) or median with interquartile range.

## Results

Patient demographics and characteristics are provided in Table 1. Institutional utilization of antiarrhythmic drugs (AADs) and oral anticoagulation on hospital discharge varied widely, with 62% (60/97) of patients discharged on class I or III AADs and 51% (49/97) discharged on oral anticoagulation.

**Table 1:** Patient Demographics and Characteristics

Parameter	NASA (N = 97)
Age, y	73 (68-76)
LVEF, %	55 (45-60)
Left atrial size, cm	4.0 (3.6-4.7)
CHA_2_DS_2_-VASc score	4.0 (3-5)
Atrial fibrillation type	
Paroxysmal	83 (86)
Nonparoxysmal (Per/LSP)	14 (14)
Duration of atrial fibrillation, y	1.0 (0-3)
Hospital LOS, d	8.0 (5-11)

Categorical variables are presented as number (percentage). Continuous variables are presented as median (interquartile range).

CHA_2_DS_2_-VASc, congestive heart failure, hypertension, age ≥75 years (doubled), diabetes, stroke (doubled), vascular disease, age 65 to 74 years, sex category (female); LOS, length of stay; LVEF, left ventricular ejection fraction; NASA, non-atriotomy surgical ablation; Per/LSP, persistent/long-standing persistent.

### Operative NASA Results

No intraoperative complications (superior vena cava, inferior vena cava, pulmonary vein, right atrial, left atrial, or left atrial appendage tear or perforation; ablation-related left atrial or left atrial appendage thrombus) were observed (0/97). No 30-day cerebrovascular events (strokes, transient ischemic attacks) or systemic embolic events occurred. Three (3/97 [3%]) patients required permanent pacemaker implantation. Postoperative atrial fibrillation occurred in 13.4% (13/97) of patients. Six patients (6/13 [46%]) resumed normal sinus rhythm with AAD use. Three patients (3/13 [23%]) with postoperative atrial fibrillation underwent cardioversion. Differences in cross-clamp time (74 + 19 minutes vs 75 + 30 minutes) and bypass time (95 + 23 minutes vs 107 + 44 minutes) between isolated CABG and CABG + NASA have previously been reported.^7^

### FFAF <30 Seconds On or Off Class I or III AADs

With a median follow-up of 12 (12-24) months, 43 patients (44% [43/97]) had CAM and 95% (41/43) demonstrated FFAF <30 seconds on or off AADs; 93% (40/43) showed FFAF <30 seconds off AADs.

### FFAF <30 Seconds On or Off ADDs With >12 Months of Follow-Up

**Figure 2:**
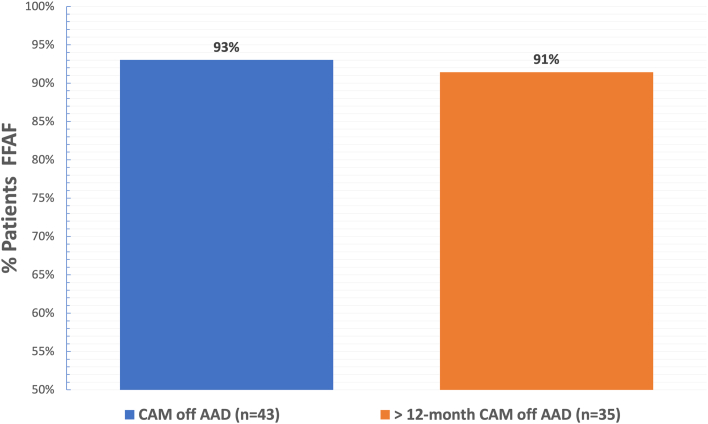
Associated rhythm outcomes for patients undergoing non-atriotomy surgical ablation. In patients who underwent continuous ambulatory monitoring (CAM), freedom from atrial fibrillation, flutter, and tachycardia (FFAF) <30 seconds off a class I or III antiarrhythmic drug (AAD) at initial and ≥12-month follow-up was 93% and 91%, respectively.

There were 35 patients (36% [35/97]) who had CAM for a minimum of 12 months (median, 12 months; interquartile range, 12-24 months), and FFAF <30 seconds on or off AADs was 94% (33/35); 91% (29/32) showed FFAF <30 seconds off AADs (Figure 2).

### Rhythm Outcome Analysis of Paroxysmal and Nonparoxysmal Atrial Fibrillation

Most patients in this study cohort had preexisting PAF (83/97 [86%]). All patients with preexisting PAF treated with NASA and observed with CAM exhibited complete FFAF <30 seconds off AADs at both initial (37/37 [100%]) and ≥12-month follow-up (30/30 [100%]). Six patients with preexisting non-PAF were treated with NASA and observed with CAM, and 50% (3/6) exhibited FFAF <30 seconds off AADs at the initial follow-up. Five patients with preexisting non-PAF were treated with NASA and observed with CAM for at least 12 months, and 40% (2/5) exhibited FFAF <30 seconds off AADs (Table 2).

**Table 2:** HRS Rhythm Outcomes Analysis of NASA-Treated Patients With Paroxysmal and Nonparoxysmal Atrial Fibrillation

Patients	FFAF Off AAD With CAM	FFAF Off AAD With CAM ≥12 Months
All patients	93% (40/43)	91% (32/35)
Paroxysmal AF	100% (37/37)	100% (30/30)
Nonparoxysmal AF	50% (3/6)	40% (2/5)

AAD, class I or III antiarrhythmic drug; AF, atrial fibrillation; CAM, continuous ambulatory monitoring; FFAF, freedom from atrial fibrillation, flutter, and tachycardia <30 seconds; HRS, Heart Rhythm Society; NASA, non-atriotomy surgical ablation.

## Comment

In this multicenter cohort study of patients with preexisting atrial fibrillation undergoing isolated CABG who received a NASA of the left atrium, we observed 4 important findings. First, most patients with preexisting atrial fibrillation undergoing isolated CABG who underwent NASA presented with PAF (86% [83/97]). Second, NASA was safely executed without any attributable intraoperative complications. Third, NASA was associated with favorable FFAF <30 seconds off class I or III AADs in all patients with PAF at ≥12-month follow-up with CAM. Fourth, NASA led to <50% FFAF in patients with non-PAF.

NASA of the left atrium (left atrial box) appears to effectively isolate pulmonary vein and left atrial posterior wall triggers likely to be responsible for PAF^9^; however, in non-PAF patients with likely left atrial posterior wall fibrosis^9^ and substrate remodeling susceptible to macroreentry, left atrial box ablation alone was less successful. A report by Zhang and coworkers,^10^ using a functional fibroblast activation protein inhibitor positron emission tomography–magnetic resonance imaging analysis, revealed that in non-PAF, significant fibrosis occurs in both the right and left atria, suggesting a likely biatrial-dependent mechanism in patients with long-standing persistent atrial fibrillation that may be inadequately treated with a left atrial box lesion alone.

NASA was associated with a 3% new permanent pacemaker insertion rate. This is less than the most recent STS registry report of 6.87% and may be attributed to the reported 5% sick sinus rate^11^ in PAF patients undergoing surgical ablation.

### Limitations

NASA techniques varied between surgeons and centers; therefore, specific guidance on NASA protocols cannot be provided. CAM was performed in <50% (43/97) of the total study cohort, and this can introduce significant surveillance bias as surgeons and centers that have established surveillance programs with CAM may provide heightened atrial fibrillation care.

### Conclusion

In a multicenter cohort study, a NASA was safe and had a favorable association with normal sinus rhythm at 1 year.
